# Haptoglobin gene diversity and incidence of uncomplicated malaria among children in Iganga, Uganda

**DOI:** 10.1186/s12936-020-03515-y

**Published:** 2020-11-26

**Authors:** Catherine N. Lwanira, Fred Kironde, Göte Swedberg

**Affiliations:** 1 School of Biomedical Sciences, College of Health Sciences, Makerere University, PO Box 7072, Kampala, Uganda; 2 Habib Medical School, Faculty of Health Sciences, Islamic University in Uganda (IUIU), Kampala Campus, Uganda; 3 Department of Medical Biochemistry and Microbiology, Uppsala University, Uppsala, Sweden; 4 Department of Biochemistry, Uganda Christian University School of Medicine, PO BOX 4, Mukono, Uganda

**Keywords:** Haptoglobin genotypes, *Plasmodium falciparum* malaria, Incidence of uncomplicated malaria

## Abstract

**Background:**

Haptoglobin (Hp) is an acute phase protein that takes part in systemic regulation of haem during *Plasmodium falciparum* infections. Numerous genotypes of haptoglobin have been reported in malaria endemic populations. In this study, the relationship between haptoglobin genotypes and incidence of uncomplicated malaria in a cohort of children living in a malaria-endemic area of Uganda was determined.

**Methods:**

This is an extension of a longitudinal study comprising of 423 children aged between six months and nine years, who were actively followed up for one year. Malaria episodes occurring in the cohort children were detected and the affected children treated with national policy drug regimen. Haptoglobin genotypes were determined by an allele-specific PCR method and their frequencies were calculated. A multivariate negative binomial regression model was used to estimate the impact of haptoglobin genotypes on incidence of uncomplicated malaria in the children’s cohort. In all statistical tests, a P–value of < 0.05 was considered as significant.

**Results:**

The prevalence of the Hp 1–1, Hp 2–1 and Hp 2–2 genotypes in the children’s cohort was 41%, 36.2% and 22.9%, respectively. The overall frequency for the Hp 1 allele was 59%, while Hp 2 allele occurred at a frequency of 41%. After adjustment of incidence rates for age, insecticide treated bed net (ITN) use and malaria history, the incidence of uncomplicated malaria for children carrying the Hp 2–2 genotype and those with the Hp 2–1 genotype was statistically similar (P = 0.41). Also, no difference in the incidence of uncomplicated malaria was observed between children carrying the Hp 1–1 genotype and those having the Hp 2–1 genotype (P = 0.84) or between Hp 2–2 Vs Hp 1–1 genotypes (P = 0.50).

**Conclusions:**

This study showed that the Hp 1–1 and Hp 2–1 genotypes each occur in nearly 4 in 10 children and the Hp 2–2 genotype occurs in 2 of every 10 children. No association with incidence of uncomplicated malaria was found. Additional studies of influence of haptoglobin genotypes on *P. falciparum* malaria severity are needed to understand the role of these genotypes in malarial protection.

## Background

Malaria is a major cause of morbidity and mortality among children below 5 years in sub-Saharan Africa [1]. The course of *Plasmodium falciparum* malaria infections involves interplay of both parasite and host factors such as host genetic variability [2]. In the blood stage of *Plasmodium* infection, merozoites multiply inside the red blood cells (RBCs) causing rupture of the RBC membrane and release of free haemoglobin (Hb) into circulation [3]. Tissue damage, inflammation, cytotoxicity [4] and host cell death [5] may follow as a result of accumulation of free haem. After 7–30 days from the time of the mosquito bite, malarial symptoms arise largely due to red blood cell rupture and the body’s inflammatory response [3]. Haptoglobin (Hp) determines the course of *Plasmodium* infections by binding the free haem produced from intravascular lysis of the RBCs. Hp is secreted by the liver following acute infection and binds to free haem forming a stable haptoglobin- haemoglobin (Hp-Hb) complex [6]. This complex is removed from circulation by binding to a cell-surface receptor (CD 163) expressed by monocytes or macrophages, then internalized and destroyed within the spleen [7]. This is needed in the control of free radical induced oxidative damage and inflammation that may follow *P. falciparum* infections [8].

In humans, Hp is encoded on the haptoglobin gene on chromosome *16q22.2* [8]*.* The gene is polymorphic, with two co-dominant alleles (*Hp 1 and Hp 2).* The Hp 1 gene encodes two subunits; α1and β of approximately 8.86 kDa and 40 kDa respectively. The β subunit shows no genetic polymorphism, while the *α*1 subunit has 2 allelic variants (IS and IF) that differ in amino acid composition and electrophoretic mobility. The Hp 2 gene encodes the α2 subunit of approximately 17.3 kDa and a portion of the β-subunit [8]. The genetic arrangement depicted generates three major phenotypes (Hp 1–1, Hp 2–1 and Hp 2–2) [8] that vary in the binding affinities for free haem in the order, Hp 1–1 > Hp 2–1 > Hp 2–2 [7]. In earlier studies, Hp genotypes were found to be associated with altered plasma Hp levels [9, 10] and malaria outcomes [11]. The Hp 2–2 genotype that leads to lowest circulating plasma Hp levels [12] was associated with lower incidence of clinical malaria in prospective cohort studies carried out among African populations [13, 14]. Yet in another cohort study, the Hp 2–2 phenotype was associated with a higher susceptibility to *P. falciparum* infection among the Dogon, but not the Fulani, ethnic tribe of Mali [15]. In other studies where increased risk of developing *P. falciparum* symptomatic malaria among children carrying the Hp 2–2 genotype was reported [16], no associations between the Hp 1 allele and malaria susceptibility was found [14, 16].

The frequency of the Hp 1 and Hp 2 genes varies considerably in different populations. The Hp 1 allele frequency ranges from as low as 0.07 in parts of India to over 0.7 in West Africa and South American populations [8]. Frequencies of the Hp 1 allele were found to be 0.52 among Hispanics, 0.55 in Blacks, 0.44 among Caucasians, 0.31 among Asians living in the American region and 0.56 in the African region [8]. In a study that examined the role of Hp polymorphisms in determining susceptibility to *P. falciparum* infection and severity of malaria among Ghanaian children, Hp1-1, Hp 2–1, and Hp 2–2 genotypes occurred in 32.4%, 54.1%, and 13.5% children, respectively [17]. Two studies carried out in malaria endemic Kenyan coast found the prevalence of Hp 1–1, Hp 2–1 and Hp 2–2 phenotypes of 45%, 41% and 14% [13] and 28.5%, 45.2% and 26.4% of the study children, respectively [18]. In Uganda, there are no studies that have documented the frequency of Hp genotypes/ phenotypes in the population. This study reports about the profile of Hp genotypes and their relationship with incidence of uncomplicated malaria among children in Iganga, Uganda.

## Methods

### Study design and setting

This study is an extension of a longitudinal study that took place in the malaria endemic district of Iganga- Mayuge in eastern Uganda [19]. From September 2008 to October 2008, a team of well-trained home visitors engaged households to systematically recruit children into a baseline malaria study. Eligible children were enrolled into the baseline study and followed up for a period of one year from November 2008 to November 2009. The study cohort was recruited from a community living within six villages of Iganga district that are in close proximity to the malaria study clinic located at Makerere University Iganga/Mayuge Demographic Surveillance Site (MaK-DSS). No interventional studies involving prophylactic anti-malarial mass treatment were undertaken in this study area at the time this study cohort was assembled. Inclusion criteria of the cohort study was as described in an earlier study [19] and followed; (1) six months to nine years of age; (2) agreement to come to the study clinic for any febrile episode or illness; (3) agreement to avoid medications administered outside the study; (4) agreement to remain in study area during the twelve months follow up; (5) absence of known chronic disease and 6) written informed consent provided by parent or guardian. Severely malnourished children (below -3z scores of the median World Health Organization (WHO) growth standards) [20] were excluded. Follow-up started when children fulfilled all of the selection criteria and were free of symptomatic malaria.

## Active case detection and estimation of malaria incidence

Study villages were divided by convenience into active (nearby) villages and passive (more remote) villages. Study personnel sought for verbal consent from parents/guardians of the children to participate in a brief demographic survey, and written informed consent was obtained before enrolment into the study. Using a standardized questionnaire, demographics and malaria indicator information was collected. After enrolling into the study, parents or guardians were instructed to bring their children to the malaria clinic based at Iganga Hospital whenever the children felt unwell. Follow-up started when children fulfilled all of the selection criteria and were free of symptomatic malaria.

Children were visited twice a week by the study field workers at convenient times of day. A standardized questionnaire was administered for collecting information regarding illnesses that had occurred since the last visit, use of health care facilities and medications used. At each visit, the tympanic temperature was recorded using a digital thermometer. When fever (tympanic temperature of ≥ 37.5 °C) or history of recent fever (within the last 24 h) was observed or reported for any study child, a rapid diagnostic test (RDT, OPTIMAL®) and microscopy of a stained blood smear were performed to screen for malaria and confirm the presence of malaria parasites, respectively. Uncomplicated malaria was confirmed using the WHO criteria that includes having any *P. falciparum* parasitaemia plus fever or a history of fever (within the past 24 h) [21]. Children found with asexual malaria parasitaemia were followed up after being administered artemisinin-based combination therapy (ACT) according to Uganda national treatment guidelines [22]. Severely ill children were referred to Iganga Hospital. The time at risk for new infection was defined as the duration of study participation excluding 14 days after each ACT-treated episode of malaria. The incidence of malaria was determined by calculating the number of malaria episodes per child over the one year of active follow up.

## Sample preparation and DNA extraction

Whole blood samples were obtained from all study children for subsequent DNA analyses. Blood samples were drawn into Ethylene Diamine Tetra Acetic acid (EDTA) anticoagulant tubes. Buffy coats were prepared from 1 to 2 ml of whole blood by differential sedimentation using phosphate buffer saline (PBS) and 2% fetal bovine serum (FBS). Genomic DNA was extracted from blood leukocytes using E.Z.N.A Blood DNA kit following the manufacturer’s protocol (Omega Bio-tek, USA). DNA samples were stored at -20 °C for subsequent genomic analysis.

## Haptoglobin genotyping

Haptoglobin genotypes were determined by allele-specific polymerase chain reaction amplification as described before [23], using primers sets listed in Table 1. This was based on determination of the polymorphic alpha (α) chain alleles, Hp1 and Hp2. Hp1 encodes the α1-1S (slow form) and 1F (fast form) chains, while Hp2 encodes the Hp α2 polypeptide chain. Primers F3 and C42 were used to amplify a 935 bp fragment of the Hp2 allele. To amplify the 1.2 kb DNA fragments in the Hp1S allele, primers C51 and S2 were used (reaction S). For amplification of the Hp1F allele, primers F3 and C72 were used in reaction F to produce DNA fragment of 1.4 kb.

**Table 1:** Primer sets for polymerase chain reaction

Reaction	Target alleles	Primer pair	Oligonucleotide sequence (5′-3′)	Product size (bp)
Reaction 2	Hp2	F3	CAGGAGTATACACCTTAAATG	935
		C42	TTACACTGGTAGCGAACCGA	
Reaction S	Hp1S	C51	GCAATGATGTCACGGATATC	1.2 kb
		S2	TTATCCACTGCTTCTCATTG	
Reaction F	Hp1F	F3	CAGGAGTATACACCTTAAATG	1.4 kb
		C72	AATTTAAAATTGGCATTTCGCC	

*bp* Base pair

## Polymerase chain reaction (PCR)

PCR amplification was carried out in a 25µL reaction containing 10 mM Tris–HCl (pH 9.0), 50 mM KCl, and 0.1% Triton X-100, 2.5 mM MgCl_2_, 200 µM of each dNTP, 0.2 µM of each primer and 1.5 units of Taq polymerase (Thermo Scientific, Inc). For each reaction, approximately 1µL of the DNA sample was used. After preheating at 95 °C for 3 min, PCR was performed with 35 cycles of heating at 94 °C for 40 s, at 58 °C for 1 min and annealing at 72 °C for 2 min. PCR products were separated by electrophoresis on 1.2% agarose gels stained with ethidium bromide. Hp genotypes were determined by observing the amplified DNA fragments under ultra violet light.

## Data management and analysis

Data were cleaned, coded and entered into Microsoft Office Access ™ 2007. Descriptive statistics, Chi-square tests and multivariate analysis were carried out using Stata 12.0 (Stata Corp, College Station, Texas, USA). Allele and genotype frequencies were calculated. The association between haptoglobin genotype and incidence of uncomplicated malaria was estimated using a multivariate negative binomial regression model. From a previous analysis in the same children’s cohort, age, malaria history and insecticide-treated bed net (ITN) were identified as independent predictors of malaria incidence [19]. These factors were included in the final multivariate analysis to determine the extent to which haptoglobin genotypes affected the incidence of uncomplicated malaria in the children’s cohort. Adjusted incidence rate ratios (aIRRs), P values and 95% confidence intervals were calculated. All statistical tests were two-tailed and P-values less than 0.05 were considered significant.

## Results

### Study population

A total of 434 cohort children were actively followed up for 1 year and the incidence of malaria (annual episodes per child) was determined. Of the 434 children, 2.5% (11/434) did not provide an adequate blood sample for subsequent analysis of DNA. The remaining 423 children were included in the host genetics studies. A majority of the study participants (96.7%) were of *Basoga* ethnic tribe. Sixty-five percent (275/423) were within the age range of 3–9 years and only 35% (148/423) were under 3 years of age. The mean age was 3.9 years (SD: ± 2.3). Slightly over half of the study participants (52.7%) were males. At recruitment, mean haemoglobin was 12 g/dL (SD: ± 1.5) {normal range = 8.8–12.5 g/dL} [24] and mean weight was 15.5 kg (SD ± 5.2). The predominant blood groups were O^+^ (39.4%) and B^+^ (30.4%).

## Malaria occurrence and indicators in the children’s cohort

A majority of the guardians of study participants (94.6%) reported that their children had experienced fever during the past 6 months preceding enrolment into the study. At enrolment to the study, approximately 40% (168 of the 423 children) had parasitaemia, with a median parasitaemia of 575 parasites/μL [inter quartile range (IQR) = 225–2750/μL]. About 88.2% of the participants’ guardians (373 / 423) reported owning and using an insecticide treated bed net (ITN) within their households. Four hundred and three participant’s guardians (95.3%) reported having ever administered an anti-malarial drug to the enrolled child. Throughout the 1-year of longitudinal follow up in this study, malaria episodes were not registered among 217 out of 423 children (51.3%). Among those who experienced malaria episodes (206 children; 48.7%) during the 1 year of follow up, the range of annual episodes per child was 1 to 9.

## Prevalence of haptoglobin genotypes in the children’s cohort

Haptoglobin genotyping was performed successfully for 398 samples by determining the presence or absence of 935 bp, 1.2 kb and 1.4 kb DNA fragments corresponding to the Hp2, Hp1S and Hp1F genotypes, respectively. The Hp1-1, Hp2-1 and Hp2-2 genotypes were found in 41%, 36.2% and 22.9% of the cohort children, respectively. The overall allele frequency for the Hp1 allele was 59%, while Hp2 allele occurred at an allele frequency of 41%. The distribution of the Hp genotypes in the study cohort is presented in Table 2.

**Table 2:** Haptoglobin genotypes in the study cohort

Hp Genotypes	Number N (%)	Genotype frequency, n (%)		Allele frequency, n (%)	
1S-1S	25 (6.3)	Hp1-1	163 (41)	Hp1	235 (59)
1S-1F	35 (8.8)	Hp2-1	144 (36.2)	Hp2	163 (41)
1F-1F	103 (25.9)	Hp2-2	91 (22.9)		
2-1S	55 (13.8)				
2-1F	89 (22.4)				
2-2	91 (22.9)				
Total	398 (100.0)	Total	398 (100.0)	Total	398 (100.0)

## Relationship between haptoglobin genotypes and incidence of uncomplicated malaria in the children’s cohort

This study is an extension of a longitudinal study carried out previously on the same cohort of children [19]. In this children’s cohort, a total of 414 new episodes of malaria were recorded during one year of longitudinal follow up. These episodes comprised of a child being unwell, having any level of parasitaemia and having a fever either at the time of visit to the study clinic or within the previous 24 h. The overall incidence of these episodes was 0.98 per child/year. Peak incidence (1.25 episodes/child/ year) occurred between the age range of 1–3 years, which was about 1.8 times the incidence for older children in the age range of 5–9 years (0.70 episodes/child/year). These episodes of ongoing or recent (past 24 h) febrile illness, that were accompanied by *P. falciparum* infection, were included in the final multivariate negative binomial regression model to determine the extent to which they were affected by the haptoglobin genotype after adjusting for other independent determinants of malaria incidence identified in an earlier study in the same children’s cohort [19]; namely age, malaria history, and ITN use.

There was no statistically significant difference in the distribution of the Hp genotypes among children who did and did not experience uncomplicated malaria throughout the year as shown in Table 3. Crude malaria incidence rates /child/year were 1.16 for the Hp 1-1genotype, 0.86 for Hp 2–1 and 1.01 for individuals carrying the Hp 2–2 genotype. Malaria incidence rates/child/year for the Hp1 and Hp2 alleles were 0.993 and 0.987, respectively. After adjusting for known determinants of malaria incidence (age, malaria history and ITN use), the incidence rates of uncomplicated malaria for children carrying the Hp 2–2 genotype and those with the Hp 2–1 genotype were statistically similar (P = 0.41). Also, no difference in the incidence of uncomplicated malaria was observed between children carrying the Hp 1–1 genotype and those having the Hp 2–1 genotype (P = 0.84) or between Hp 2–2 Vs Hp 1–1 genotypes (P = 0.50) as shown in Table 4.

**Table 3:** Comparison of the genotype frequencies for children who did and did not experience uncomplicated malaria

Hp genotype	Malaria episodes		Total	P-value
	YES	NO		
Genotype frequency (%)				
Hp 1–1				
Others	118 (50.2)	117 (49.8)	235	
Hp 1–1	82 (50.3)	81 (49.7)	163	0.985
Hp 2–1				
Others	129 (50.8)	125 (49.2)	254	
Hp 2–1	71 (49.3)	73 (50.7)	144	0.490
Hp 2–2				
Others	152 (49.5)	155 (50.5)	307	
Hp 2–2	48 (52.7)	43 (47.3)	91	0.588

Others- Sum of all the other Hp genotypes

**Table 4:** Effect of host Hp genotype on incidence of malaria

Hp genotype	Malaria		No. of new episodes	Adjusted incidence rate ratio	P-value	95%CI
	No	Yes				
Hp 2–1	73	71	137	Reference	–	–
Hp 1–1 Hp 2–2 Hp 1–1	81 43 81	82 48 82	168 95 168	1.03 1.16 Reference	0.839 0.405 –	0.760–1.403 0.818–1.644 –
Hp 2–1	73	71	137	0.97	0.839	0.713–1.317
Hp 2–2	43	48	95	1.12	0.501	0.800–1.579

## Discussion

Haptoglobin genotypes, Hp1-1, Hp2-1 and Hp2-2 were found in 41%, 36.2% and 22.9% of the cohort children, respectively. The overall allele frequency was 59% for the Hp1 allele and 41% for the Hp2 allele. However, no association with incidence of uncomplicated malaria was found. The present findings differ from results of prospective cohort studies carried out among Kenyan children, in which the Hp 2–2 genotype was associated with lower incidence of clinical malaria [13, 14]. In a different cohort study that was carried out among two ethnically different populations of Mali, the Hp2-2 phenotype was found to be associated with a higher susceptibility to *P. falciparum* infection in Dogon, but not in Fulani tribe [15]. The findings of the present study and those of earlier studies show variability in the influence of Hp genotypes on malaria susceptibility among the populations studied. The observable differences in the associations reported may be partly explained by the epistatic associations between the Hp genotypes and other genetic markers. However, the present study only assessed the main Hp genotypes. Other Hp genotypes and promoter polymorphisms that could affect plasma Hp levels were not investigated.

In addition, Hp genotypes have been shown to influence plasma Hp levels [9, 10] needed in systemic regulation of haem and prevention of haem-induced oxidative tissue damage during *P. falciparum* infections [8, 25]. This may be particularly important in controlling severe disease. Thus, it is possible that the Hp genotypes may have a greater role in determining malaria severity rather than malaria incidence. However, in the present study, only incidence of uncomplicated malaria was measured. Severity of infection was not assessed. The influence of Hp genotypes on severe malaria has been reported in a few case–control studies [11, 17]. Additional studies of influence of haptoglobin genotypes on *P. falciparum* malaria severity are needed to further understand the role of these genotypes in malarial protection.

The Hp1 allele was present at an allele frequency of 59%, yet no influence on the incidence of uncomplicated malaria by the Hp1 allele was observed. This is also in line with some earlier studies that found no clear associations between the Hp 1 allele and malaria susceptibility [14, 16]. Instead, the Hp1-1 phenotype was associated with higher levels of tumour necrosis factor (TNF) and interferon gamma (IFN- γ) in an earlier study [15], suggesting that this allele may have been maintained by protection from other infections [14]. Since only a handful of studies have investigated the role of Hp genotypes in determining malaria susceptibility yet providing equivocal findings, more extensive studies in different populations are needed to confirm these associations.

## Conclusions

Haptoglobin genotypes, Hp1-1, Hp2-1 and Hp2-2 were found in 41%, 36.2% and 22.9% of the cohort children, respectively. The overall allele frequency was 59% for the Hp1 allele and 41% for the Hp2 allele. Association of Hp genotypes with incidence of uncomplicated malaria was not observed. This lack of detectible association with uncomplicated malaria suggests that these alleles may have been maintained by protection from other infections. Similar studies in different settings are needed to confirm these findings.

## Data Availability

The clinical and laboratory datasets used and /or analysed during this study are available from the corresponding author upon request.

## Abbreviations

RBC: Red blood cell
ROS: Reactive oxygen species
Hb: Haemoglobin
Hp: Haptoglobin
CD 163: Cluster of differentiation 163
RDT: Rapid diagnostic test
ACT: Artemisinin-based combination therapy
IFN- γ: Interferon-gamma
TNF: Tumour necrosis factor
HIV: Human immunodeficiency virus
WHO: World Health Organization
EIR: Entomological inoculation rate
EDTA: Ethylene diamine tetraacetic acid
DNA: Deoxyribonucleic acid
PCR: Polymerase chain reaction
aIRR: Adjusted incident rate ratio
IQR: Inter quartile range
SD: Standard deviation
ITN: Insecticide-treated bed net
